# Living cell imaging and Rac1-GTP levels of CXCL12-treated migrating neural progenitor cells in stripe assay

**DOI:** 10.1016/j.dib.2015.09.048

**Published:** 2015-10-28

**Authors:** Min Zhang, Aihong Song, Siqiang Lai, Lisha Qiu, Yunlong Huang, Qiang Chen, Bing Zhu, Dongsheng Xu, Jialin C. Zheng

**Affiliations:** a Center for Translational Neurodegeneration and Regenerative Therapy, Shanghai Tenth People’s Hospital affiliated to Tongji University School of Medicine, Shanghai, China; b Department of Pharmacology & Experimental Neuroscience, University of Nebraska Medical Center, Omaha, NE 68198, United States; c Department of Pathology and Microbiology, University of Nebraska Medical Center, Omaha, NE 68198, United States

## Abstract

This data article contains three figures and three videos related to the research article entitled “Applications of Stripe Assay in the Study of CXCL12-mediated Neural Progenitor Cell Migration and Polarization” Zhang et al. (2015) [1], which uses stripe assay to study mouse neural progenitor cell (NPC) migration and polarization. The current article describes the neurosphere method used to culture NPCs. NPCs in neurospheres and monolayer were characterized using immunocytochemistry method with antibodies against two classic NPC markers: nestin and SOX2. The article also describes method to obtain sufficient protein lysates from NPCs in the stripe assay. When protein lysates were subjected to Rac1 affinity precipitation, Rac1-GTP was detected in the pull-down samples. In addition, the articles provides live cell imaging data to better understand CXCL12-mediated cellular migration and polarization.

Specifications table.

**Table:** 

Subject area	*Biology*
More specific subject area	*Assays to study cellular migration and polarization*
Type of data	*Figure and video*
How data was acquired	*Zeiss 710 confocal microscope and Zeiss Live cell Imaging System*
Data format	*Raw*
Experimental factors	*CXCL12 stripe and control stripe*
Experimental features	*NPCs were first characterized in immunocytochemistry and then subjected to stripe assays for Rac1-GTP affinity precipitation and live cell imaging.*
Data source location	*Shanghai, China*
Data accessibility	*data is with this article*

1. The NPCs express both nestin and SOX2. The data can be referenced when identifying NPCs in the cultures.
2. Rac1 is an important signaling intermediate for migration and polarization. Sufficient amount of protein lysates can be acquired from stripe assay for Rac1-GTP pull-down. Detection of Rac1-GTP in the pull-down samples will be a valuable bench mark for future studies aiming to identify cell signaling during migration and polarization.
3. Stripe assay can be used to observe NPCs׳ migration and polarization toward CXCL12 stripes by live cell imaging. This data significantly extends the applications of stripe assay in cell biology.

## 1 Data

**Fig. 1:**
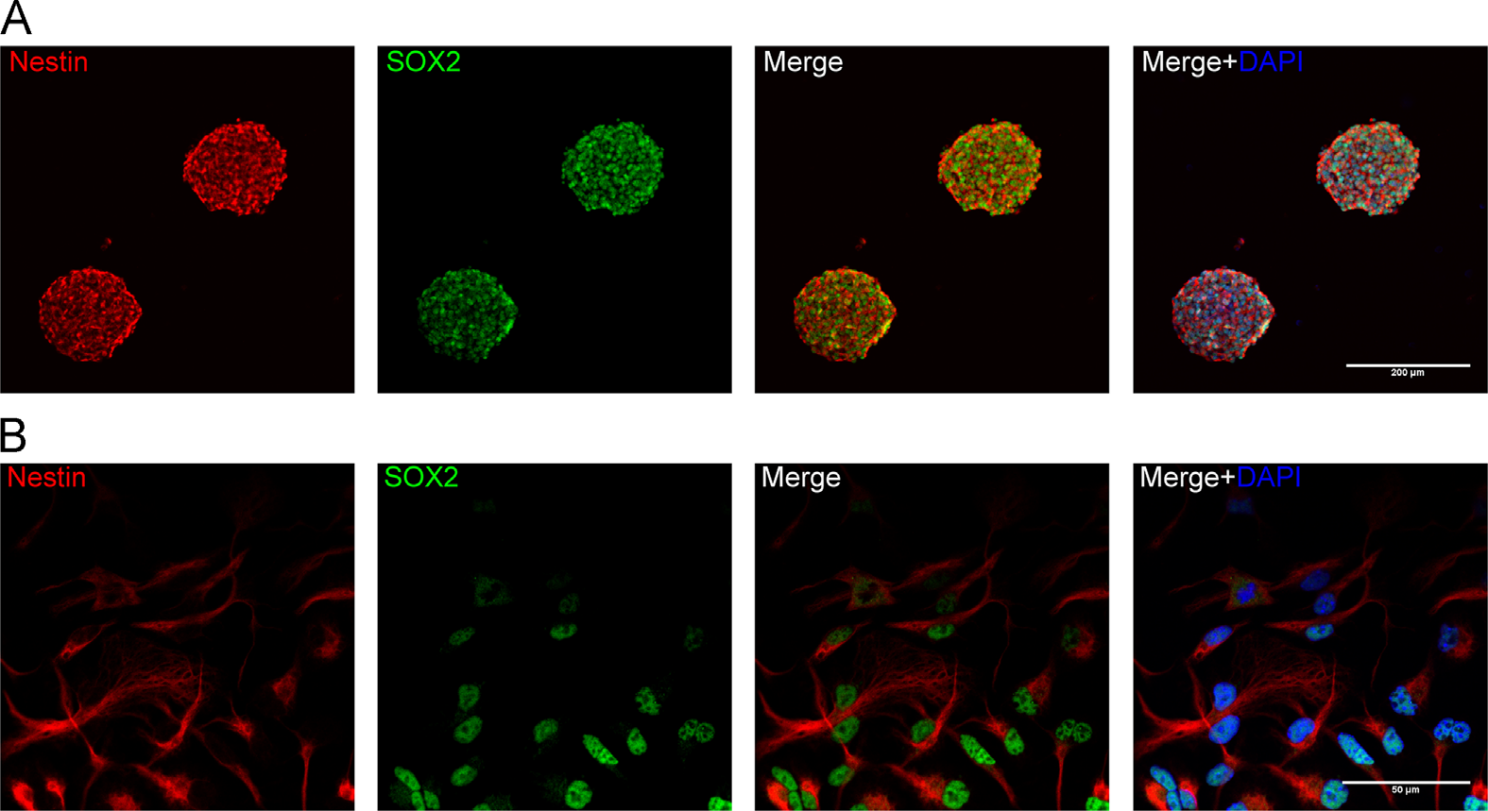
Characterization of mouse NPCs in immunocytochemistry using antibodies against nestin and SOX2. Mouse NPC neurosphere (A) and adherent (B) cultures were fixed and stained with nestin (red), SOX2 (green), and DAPI (blue). Merge and Merge+ are merged pictures of nestin/SOX2 and nestin/SOX2/DAPI, respectively. Results are representative of three independent experiments. Scale bar: A: 200 µm, B: 50 µm (For interpretation of the references to color in this figure legend, the reader is referred to the web version of this article).

**Fig. 2:**
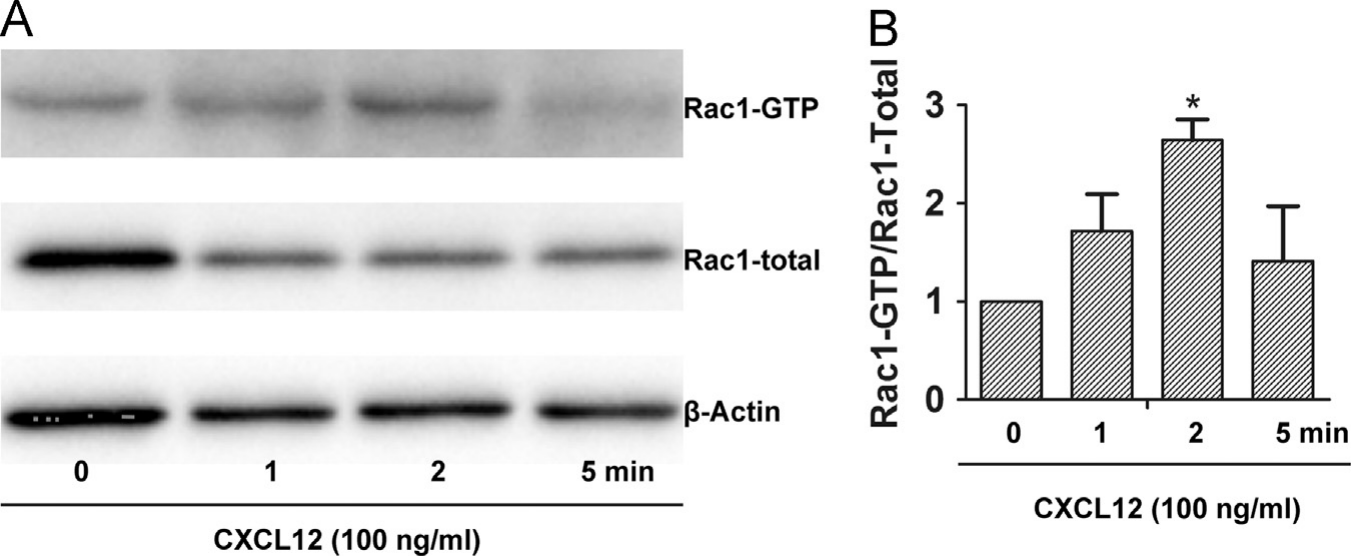
CXCL12-mediated NPC Rac1 activation in stripe assays. Mouse NPCs (1×10^7^/ml) were seeded on Poly-D-lysine dishes printed with either BSA stripes or BSA CXCL12 stripes. A) At the indicated time points, whole cell lysates of NPCs were collected and subjected to affinity precipitation for Rac1-GTP. Levels of Rac1-GTP and total Rac1 were analyzed by Western blot. Actin in whole cell lysates was used as loading control. B) Levels of Rac1-GTP were normalized as a ratio to total Rac1 and shown as fold change relative to the untreated controls. * denotes *p*<0.05, compared with untreated control.

**Fig. 3:**
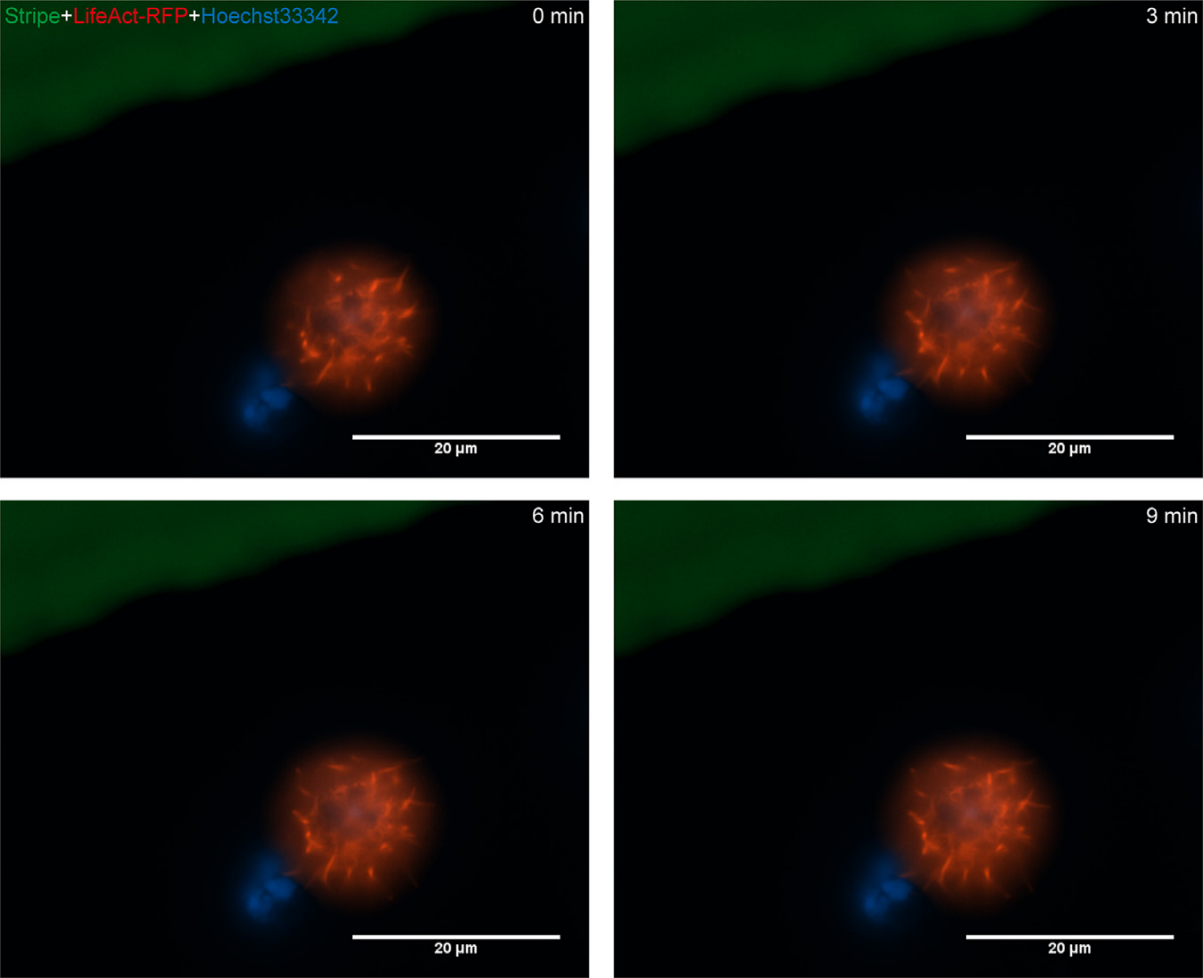
Live Cell images of NPCs in stripe assay with control BSA stripes. Mouse NPCs were seeded on cover glasses printed with FITC-BSA stripes (green) after transfection with LifeAct-RFP and nuclear DNA stained with Hoecst33342. During 10 min in observation, cells remained unpolarized and formed only filopodia. Scale bar: 20 µm. (For interpretation of the references to color in this figure legend, the reader is referred to the web version of this article.)

To better understand functional impacts of CXCL12 on NPC biology, NPCs were isolated from E13.5 mouse forebrains and enriched through neurosphere cultures [1]. A majority of mouse NPCs in neurospheres and monolayer (adherent) cultures expressed both nestin and SOX2 (Fig. 1). Because Nestin and SOX2 are markers for NPCs [2], [3], the positive staining of nestin and SOX2 in our cultures suggests that these cells are indeed NPCs. Number of living cells are critical to study mechanisms of cellular migration and polarization. Stripe assay could obtain directional migrating cells for protein analysis. Levels of Rac1-GTP increased at 2 min by CXCL12 in the stripe assay (Fig. 2), suggesting that Rac1 is activated in the NPCs by CXCL12 stripes in the assay. Migration and polarization of cells are integrated processes of cell movement. In live cell imaging, NPCs were recorded in stripe assays migrating to CXCL12 stripes in 5 h (Video1). More importantly, NPCs polarization was also recorded through live cell imaging in stripe assays (Video 2), but not control BSA stripes (Video 3), induced NPC polarization (Fig. 3).

Supplementary material related to this article can be found online at 10.1016/j.dib.2015.09.048.

The following is the Supplementary material related to this article Video 1, Video 2, Video 3.

Video 1 **A NPC migrated towards CXCL12 stripe**. Live cell imaging was setup to observe CXCL12-mediated NPC migration. Images were captured every 30 min. The total recording time was 5.5 h. Scale bar: 10 μm.

Video 2 **A NPC was polarized towards CXCL12 stripe**. Live cell imaging was setup to observe CXCL12-mediated NPC polarization. Images were captured every 3 min. The total recording time is 1 h and 27 min. Scale bar: 20 μm.

Video 3 **A NPC was not polarized towards BSA stripe**. Live cell imaging was setup to observe NPC polarization in stripe assay with only control BSA stripes. Images were captured pictures every 3 min. The total recording time is 1 h and 27 min. Scale bar: 20 μm.

## 2 Experimental design, materials and methods

### 2.1 Characterizationof mouse NPCs

Mouse NPCs were fixed using 4% paraformaldehyde (PFA), and permeabilized with 0.4% triton-X in PBS. After blocked by 1% BSA in PBS, mNPCs were incubated with primary antibodies (mouse anti-nestin, 1:200, Millipore; rabbit anti-SOX2, 1:500, Cell Signal Technology) overnight. Cultures were then washed and incubated with secondary antibodies (Alexa Fluor 568 goat anti-mouse IgG, 1:500, Invitrogen; Alexa Fluor 488 goat anti-rabbit IgG, 1:500, Invitrogen) for one hour at room temperature. Nuclear DNA was labeled with 4′, 6-diamidino-2-phenylindole (DAPI; Sigma-Aldrich) for 2 min after the secondary antibody at room temperature. Cover slips were mounted on glass slides with mounting medium (Sigma-Aldrich). Triple immunostaining was examined by a Zeiss 710 confocal microscope.

### 2.2 Pull-down assay

Active Rac1 pull-down and detection kit (Thermo scientific) was utilized for the detection of Rac1-GTP level. The assay was performed according to the manufacturer’s instruction. The kit provides a GST-fusion protein containing the p21-binding domain (PBD) of human p21-activated protein kinase 1 (Pak1) along with glutathione agarose resin to specifically pull down active Rac1. Briefly, the cell lysates were incubated with the GST-Pak1 beads. Levels of bead-bound GTP-Rac1 and total Rac1 proteins were analyzed by immunoblot using an anti-Rac1 antibody (1:1000; Thermo scientific) and β-actin (Sigma) was as internal reference.

### 2.3 Living imaging experiment

After transfection of LiveAct-RFP and nuclear DNA stained with Hoechst33342, NPCs were dissociated with accutase (Gibco) into single cells. The stripe-coated dishes were seeded with dissociated NPCs to study living cell migration or polarization. Images were taken by times-series of Zeiss Live cell Imaging System.
